# Characterization and *ex vivo* modelling of endodontic infections from the Arabian Gulf region

**DOI:** 10.1111/iej.14227

**Published:** 2025-03-26

**Authors:** Rania Nassar, Mohannad Nassar, Lobna Mohamed, Abiola Senok, David Williams

**Affiliations:** ^1^ College of Medicine Mohammed Bin Rashid University of Medicine and Health Sciences, Dubai Health Dubai UAE; ^2^ School of Dentistry College of Biomedical and Life Sciences, Cardiff University Cardiff UK; ^3^ Department of Restorative Dentistry College of Dental Medicine, University of Sharjah Sharjah UAE

**Keywords:** biofilms, endodontic infections, ex vivo model, metagenomics, polymicrobial biofilms, shotgun metagenomic sequencing

## Abstract

**Aim:**

The microbiota of endodontic infections in patients from the Arabian Gulf region (AGR) is largely unexplored. While research in different global regions has investigated the microbial composition of such infections, studies using shotgun metagenomic sequencing (SMS) alongside culture‐dependent techniques (CDT) are limited. There are also few *in vitro* biofilm models that reflect the microbial profiles of endodontic infections. Therefore, by employing SMS and CDT, this research aimed to explore compositional and functional microbial profiles of endodontic infections from the AGR. The research also sought to develop *ex vivo* biofilms directly from endodontic infection samples.

**Methodology:**

SMS and CDT were used to analyze 32 root canal samples from necrotic pulp. Patients' samples were categorized into two cohorts: symptomatic (*n* = 19) and asymptomatic (*n* = 13). Samples underwent sequencing followed by bioinformatic analysis to investigate microbial composition, resistome, virulome, and functional differences. Two representative samples (8R, 15R) were selected to develop *ex vivo* biofilms on hydroxyapatite coupons. Similarity between inoculum and developed biofilms was assessed using SMS and CDT. The reproducibility of developed biofilms was assessed based on microbial composition and relative abundance at the species level using correlation coefficient analysis.

**Results:**

Endodontic samples had high bacterial diversity, with a total of 366 bacterial species detected across the two cohorts. Several antibiotic resistance (*n* = 59) and virulence (*n* = 82) genes were identified, with no significant differences between the cohorts. CDT identified 28 bacterial species, with 71.4% of the isolated bacteria having phenotypic resistance to clinically relevant antibiotics. SMS showed that the *ex vivo* biofilms were polymicrobial. Biofilm derived from sample 15R had 9 species and was dominated by *Enterococcus faecalis*, while sample 8R had 12 species and was dominated by *Streptococcus mutans*. Pearson correlation analysis demonstrated a significant positive correlation between biological biofilm replicates, confirming the reproducibility of biofilm formation.

**Conclusions:**

There was high bacterial diversity in root canal samples from necrotic pulp. Samples were shown to contain antibiotic resistance and virulence genes, with no differences evident between symptomatic and asymptomatic infections. A high number of isolated bacteria were resistant to clinically used antibiotics. *Ex vivo* biofilm models from clinical samples were successfully developed and reproducibly reflected a polymicrobial composition.

## INTRODUCTION

Apical periodontitis is a sequel to endodontic infection that causes inflammation and destruction of periradicular tissues (Nair, 2004). The microbiota of endodontic infection is comprised of heterogeneous polymicrobial communities (Jhajharia et al., 2015; Neelakantan et al., 2017) of different microorganisms, including fungi, archaea, viruses and bacteria. Bacteria are considered the most predominant and diverse microbial type in these infections (Figdor & Sundqvist, 2007; Li et al., 2009; Saboia‐Dantas et al., 2007; Siqueira Jr. & Sen, 2004).

Characterization of the microbiota in endodontic infections is required for a better understanding of disease aetiology, pathogenesis and microbial interactions. Such knowledge can provide the basis for subsequent development of therapeutic and preventive strategies to manage endodontic infection.

The microbial composition and interactions of polymicrobial communities in endodontic infections define the ‘aggressiveness’ of the infection and the severity of subsequent host inflammatory responses and symptoms (Siqueira & Rôças, 2013). Studies have shown that the microbial composition of symptomatic and asymptomatic endodontic infections differs significantly (Rôças et al., 2011; Sakamoto et al., 2006; Santos et al., 2011). It is also believed that the composition of endodontic biofilms varies with geographical location (Baumgartner et al., 2004; Machado de Oliveira et al., 2007; Siqueira Jr. et al., 2008). However, these previous investigations have primarily focused on culture‐dependent techniques, targeted molecular methods, and 16S rRNA gene sequencing (Anderson et al., 2012; Rôças et al., 2008, 2011; Siqueira Jr. et al., 2008; Tennert et al., 2014; Wong et al., 2021). There is a paucity of research using metagenomic sequencing, namely shotgun metagenomic, to investigate compositional and functional profiles of endodontic infections. Furthermore, there are no studies from the Arabian Gulf Region that characterize the microbiota of endodontic infections. This current research aimed to address this knowledge gap.

Developing *in vitro* oral biofilm models that better reflect the *in vivo* environment is an active area of research. Such models would help facilitate experimental approaches to increase our knowledge of infection processes and their management. Undeniably, endodontics is an area that lacks a standardized biofilm model for ‘bench to bedside’ translational research (Swimberghe et al., 2019). There is pressing need for a biofilm model system with sufficient complexity to capture the polymicrobial nature of endodontic infections (Swimberghe et al., 2019). Several studies have used oral samples such as saliva as an inoculum to develop *in vitro* oral biofilms (Edlund et al., 2013; Simon‐Soro et al., 2022) and has been suggested that such ‘*ex vivo* models’ might better mirror the *in vivo* situation compared to the use of preselected bacterial species (Simon‐Soro et al., 2022). Therefore, the second objective of this research was to use endodontic infection‐derived samples to directly ‘seed’ development of *ex vivo* biofilms.

## MATERIALS AND METHODS

Ethical approval for the study design and protocols was reviewed and approved by the Research Ethics Committee of the University of Sharjah (reference number REC‐21‐09‐20‐03). This manuscript was written in compliance with Preferred Reporting Items for Laboratory studies in Endodontology (PRILE) 2021 guidelines (Figure S1).

### Clinical data

Dental examination was undertaken, and clinical features were recorded for each participant. All patients provided informed consent for participation in this study. Adult patients (18–68 years old) with no history of serious systemic disorders were deemed eligible for inclusion. The sampled tooth had to be a carious mature permanent tooth with necrotic pulp and clinical and/or radiographic evidence of periradicular disease with no history of trauma. In addition, the pulp chamber had to have no visible communication with the oral environment, as well as no periodontal pockets deeper than 3.5 mm. Patients were excluded if tooth isolation was not possible using a rubber dam or if the tooth had a poor prognosis. Excluded patients were those with systemic disease, had been in receipt of antibiotic therapy in the previous 90 days, had required antibiotic prophylaxis for the intended dental procedure or were pregnant or lactating. Cases were divided into symptomatic or asymptomatic depending on the presence of periradicular originating pain, including tenderness to percussion or palpation.

### Endodontic sample collection

All chemicals, unless otherwise stated, were purchased from Sigma‐Aldrich. The aseptic method used for root canal sampling was adopted from Vianna et al. (2005). Briefly, teeth were isolated by a rubber dam, and the operative field was disinfected using 30% hydrogen peroxide (H_2_O_2_) until no further effervescence was evident. This was followed by the application of 2.5% sodium hypochlorite (NaOCl) for 1 min, which was then deactivated by 5% sodium thiosulfate (Na_2_S_2_O_3_) for 1 min. The bulk of the caries and weakened tooth structure was removed during the first stage of access cavity preparation under sterile saline using a sterile diamond bur without exposure of the pulp chamber. The disinfection protocol was repeated, and a swab sample was obtained from the internal wall of the access cavity to check sterility. The pulp chamber was entered using a new sterile diamond bur, as described above. The length of each root canal was determined based on a pre‐operative radiographic image. Sterile saline was introduced in the canal without overflowing and was agitated using a sterile K‐file number 10 (Dentsply Maillefer, Baillagues, Switzerland) followed by placing a sterile paper point (Dentsply Maillefer) to the predetermined canal length and kept in position for 1 min to collect the sample. The paper point was immediately transferred to a 2 mL microcentrifuge tube containing sterilized thioglycolate medium. Samples were stored immediately at −80°C until further use.

### Sample processing

Patients' samples and two negative control samples (that contained sterile thioglycolate media and sterile paper points) were prepared for sequencing. Samples were vortex mixed for 1 min at high speed. One mL of DNA/RNA shield™ solution (Zymo Research, USA) was added to a 500 μL aliquot of sample and vortex mixed. This preparation was then used for sequencing. The remaining sample aliquots were used for culture‐dependent microbial identification and development of *ex vivo* biofilms.

#### Microbial composition and antimicrobial susceptibility profiling

Patient samples were cultured on blood agar, CDC blood agar, bile esculin agar, Sabouraud dextrose agar (SDA), Sabouraud brain heart infusion with gentamycin and chloramphenicol (SABHI) agar, and Schaedler kanamycin and vancomycin agar with 5% sheep blood (Schaedler‐KV). CDC blood agar and Schaedler‐KV agars were incubated anaerobically at 37°C. All other agars were incubated aerobically with or without 5% CO_2_ at 37°C. Agar plates were incubated for up to 7 days, or for up to two weeks in the case of anaerobic plates. After incubation, distinct colonies were sub‐cultured on blood agar to obtain pure isolated colonies for subsequent identification and antibiotic susceptibility profiling. Colonies grown under anaerobic conditions were sub‐cultured on blood agar or CDC blood agar and incubated under both aerobic and anaerobic conditions, respectively, to determine aerotolerance. Samples yielding no growth were enriched by adding 100 μL of the original sample aliquot to 4 mL of tryptone soya broth (TSB) and thioglycolate fluid broth and incubated at 37°C under both aerobic and anaerobic conditions for up to 7 days. Samples showing turbidity were repeat cultured as described above. The identity and antibiotic susceptibility profile of isolated colonies were determined using the automated VITEK 2 system (bioMérieux, France), as described by the manufacturer. Briefly, the bacterial suspension for identification and susceptibility card inoculation was prepared using a sterile disposable loop to transfer and suspend colonies of pure culture in 3.0 mL of sterile saline. The turbidity of the suspension was measured using the DensiChek™ (bioMérieux, USA) and adjusted to that recommended by the manufacturer. Suitable identification and susceptibility Vitek cards were chosen based on prior Gram staining results.

#### Metagenomic analysis and bioinformatic analysis

Metagenomic analysis using next‐generation shotgun sequencing was performed by CosmosID (CosmosID Inc., Germantown, MD). DNA was extracted using a PowerSoil Pro kit (Qiagen) according to the manufacturer's protocol. DNA was quantified using a GloMax Plate Reader System (Promega) with the QuantiFluor® dsDNA System (Promega) chemistry. DNA libraries were prepared using the Nextera XT DNA Library Preparation Kit (Illumina) and IDT Unique Dual Indexes with a total DNA input of 1 ng. Genomic DNA was fragmented using a proportional amount of Illumina Nextera XT fragmentation enzyme. Unique dual indexes were added to each sample followed by 15 PCR cycles to construct libraries. DNA libraries were purified using AMpure magnetic Beads (Beckman Coulter) and eluted in QIAGEN EB buffer. DNA libraries were quantified using a Qubit 4 fluorometer and Qubit™ dsDNA HS Assay Kit. Libraries were then sequenced using an Illumina NovaSeq S4 platform 2 × 150 bp at CosmosID Laboratories. Unassembled sequencing reads were uploaded and analyzed by the CosmosID‐HUB Microbiome Platform (https://www.cosmosid.com/), as previously described for multi‐kingdom microbiome analysis and quantification of the relative abundance of organisms (Lax et al., 2014; Ponnusamy et al., 2016; Roy et al., 2018). Similarly, community resistome and virulome were also identified by querying unassembled sequence reads against the CosmosID curated antibiotic resistance and virulence‐associated gene databases. For metagenomic functional profiling, the UniRef90 database was used to identify functional proteins. Identified hits were annotated with MetaCyc reactions (metabolic enzymes) to reconstruct and quantify complete pathways in the microbial community sample.

#### Aerotolerance categorizing

After species identification, aerotolerance was manually identified based on the BacDive database (https://bacdive.dsmz.de).

### Biofilm formation from patients' samples

Patients' samples were used as the seeding inoculum to grow biofilms anaerobically on hydroxyapatite (HA) coupons. One clinical sample (designated 8R) was used as a representative of symptomatic apical periodontitis (SAP), while another sample (designated 15R) represented asymptomatic apical periodontitis (AAP).

To grow biofilms, sterile HA coupons were aseptically placed in sterile petri dishes. One hundred μL of the sample was added to 10 mL of 30 g/L TSB that had been pre‐reduced for 24 h in an anaerobic chamber. The suspension was vortex mixed and added to a petri dish containing HA coupons. This was then incubated anaerobically for 24 h at 37°C. After incubation, the HA coupons were aseptically transferred to a new petri dish containing 10 mL pre‐reduced 3 g/L TSB and incubated anaerobically for 24 h at 37°C. The HA coupons were then aseptically transferred to a new petri dish containing 10 mL of pre‐reduced 1 g/L TSB and incubated anaerobically for a further 24 h at 37°C. The sequential reduction in TSB concentration was done to represent gradual nutrient deprivation that occurs in endodontic infections as the infection progresses.

#### Metagenomic analysis and cultivable bacteria of *ex vivo* biofilms

After biofilm formation, the coupons were washed with PBS to remove loose or unbound microorganisms. Coupons were then swabbed using a Zymo swabbing kit (Zymo Research). Samples were then sent to CosmosID for sequencing, as described above.

To identify and quantify cultured bacteria from *ex vivo* biofilms, the biofilms on coupons were washed with PBS to remove unattached bacteria. Coupons were then transferred to 4 mL of PBS and colony‐forming units (CFUs) were enumerated as previously described (Nassar et al., 2023). Bacterial identification was achieved using the VITEK 2 system as described above.

#### Microscopic analysis of biofilms

Biofilms were developed on HA coupons as described above and imaged by scanning electron microscopy (SEM). Briefly, after incubation, HA coupons were immersed in PBS to remove unbound bacteria. HA coupons were then placed in 2.5% glutaraldehyde and left overnight in a fume hood. Coupons were then washed twice in PBS. Biofilms were dehydrated by sequential transfer in 2 mL of an ethanol solution series (50%, 70%, 85%, 95%, and 100%) for 10 min at each concentration. Dehydration with 100% ethanol was repeated twice. After the final dehydration, coupons were left to dry overnight. The samples were then sputter coated with gold and imaged using a Tescan VAGA SEM system at 5–10 kV.

### Statistical analysis

Chao1, Simpson and Shannon diversity indices using the CosmosID‐HUB Microbiome platform were used to assess alpha diversity (richness and evenness) of samples. Beta diversity between the two patient cohorts was measured by the Bray–Curtis index to estimate compositional differences between communities. The resulting Bray‐Curtis distance matrices were ordinated using Principal Coordinate Analysis (PCoA) to visualize the dissimilarity between samples. Linear discriminant analysis (LDA) effect size (LEfSe) analysis was performed to determine discriminative microbial features between the compared groups. LEfSe was calculated with Kruskal–Wallis (*p* < .05), Wilcoxon (*p* < .05) and LDA threshold scores >2. All these analyses were conducted using the CosmosID‐HUB Microbiome Platform. Pearson correlation coefficient analysis was performed using GraphPad Prism 9.4 to assess the consistency between biological biofilm replicates and to evaluate the relationship between the microbial composition of the inoculum and the developed biofilms. The resulting data was presented in a heatmap to highlight correlations. A *p* value < .05 was considered significant.

## RESULTS

### Study participant age and gender

Thirty‐two samples were collected from patients who attended University Dental Hospital Sharjah for endodontic treatment between November 2021 and March 2022. The mean age of participants was 38 years. Table S1 summarizes the demographic and clinical information of the sampled patients.

### Cultivable bacteria and phenotypic antibiotic susceptibility profile

Twenty‐eight bacterial species were identified by culture‐dependent approaches (Table 1). Seven samples yielded no growth on any of the agars. The number of cultured bacterial species from samples ranged between 1 and 6 species. Obligate anaerobic bacteria were isolated from 15.6% of the study samples, while the rate was 75% for facultative anaerobes. Both symptomatic and asymptomatic cases yielded facultative anaerobic Gram‐positive cocci (16 out of 32) and bacilli (20 out of 32), obligate anaerobic Gram‐positive cocci (4 out of 32) and obligate anaerobic Gram‐negative bacilli (2 out of 32). *Lactobacillus* spp., *Streptococcus* spp. and *Actinomyces* spp. were isolated from 40.6%, 37.5% and 28.1% of the study samples, respectively. *Enterococcus* spp., *Prevotella* spp. and *Peptoniphilus* spp. were all isolated at a rate of 6.3%. Meanwhile, *Veillonella* spp., *Parvimonas* spp., *Peptostreptococcus* spp. and *Staphylococcus saccharolyticus* were only isolated at a rate of 3.1%.

**TABLE 1:** Cultivable bacteria isolated from patient samples and identified using VITEK 2.

Classification by Gram reaction/morphology/aerotolerance	Bacteria	Sample ID																															
		Asymptomatic													Symptomatic																		
		1R	7R	11R	15R	17R	18R	19R	21R	22R	23R	24R	25R	28R	2R	3R	4R	5R	6R	8R	9R	10R	12R	13R	14R	16R	20R	26R	27R	29R	30R	31R	32R
Facultative anaerobic Gram‐positive bacilli	*Actinomyces naeslundii*																																
	*Actinomyces odontolyticus*																																
	*Atopobium vaginae*																																
	*Eggerthia catenaformis*																																
	*Eubacterium limosum*																																
	*Lactobacillus acidophilus*																																
	*Lactobacillus casei*																																
	*Lactobacillus fermentum*																																
	*Lactobacillus gasseri*																																
	*Lactobacillus hilgardii*																																
	*Lactobacillus paracasei*																																
	*Lactobacillus plantarum*																																
	*Propionibacterium propionicum*																																
Facultative anaerobic Gram‐positive cocci	*Enterococcus casseliflavus*																																
	*Enterococcus faecalis*																																
	*Gemmella bergeri*																																
	*Streptococcus anginosus*																																
	*Streptococcus gordonii*																																
	*Streptococcus mitis/oralis*																																
	*Streptococcus salivarius*																																
	*Streptococcus sanguinis*																																
Obligate anaerobic Gram‐positive cocci	*Veillonella* spp																																
	*Parvimonas micra*																																
	*Peptoniphilus asaccharolyticus*																																
	*Peptostreptococcus anaerobius*																																
	*Staphylococcus saccharolyticus*																																
	*Unidentified*																																
Obligate anaerobic Gram‐negative bacilli	*Prevotella disiens*																																

*Note*: The orange filled box indicates bacteria were isolated.

Table 2 shows the antibiotic susceptibility of selected bacterial species from patient samples. Ten species (*n* = 14, 71.4%) were resistant to at least one of the tested antibiotics. The three tested *Enterococcus* spp. were resistant to Quinupristin/Dalfopristin. Both *Enterococcus casseliflavus* (isolated from two different patients) were resistant to vancomycin. All tested species were sensitive to tigecycline or linezolid. Only *Streptococcus salivarius*, isolated from patient 1R, was resistant to chloramphenicol. Of the tested species, 35.7% were resistant to tetracycline and erythromycin. Except for one sample (14R), all isolated species that were resistant to erythromycin also had resistance to tetracycline. Five out of 14 tested species (35.7%) had intermediate tolerance to ampicillin.

**TABLE 2:** Clinical isolates' identities and their susceptibility profiles to selected antibiotics using VITEK 2.

ID	Organism	Ampicillin		Erythromycin		Linezolid		Tetracycline		Tigecycline		Quinupristin /Dalfopristin		Vancomycin		Chloramphenicol	
		MIC	Inter	MIC	Inter	MIC	Inter	MIC	Inter	MIC	Inter	MIC	Inter	MIC	Inter	MIC	Inter
1R	*S. salivarius*	4	I	≤0.12	S	≤2	S	2	S	–	–	–	–	1	S	≥16	R
14R	*S. salivarius*	4	I	≥8	R	≤2	S	1	S	–	–	–	–	1	S	4	S
8R	*S. anginosus*	≤0.25	S	≤0.12	S	≤2	S	≥16	R	≤0.06	S	–	–	0.5	S	4	S
32R	*S. anginosus*	≤0.25	S	2	R	≤2	S	≥16	R	≤0.12	S	–	–	0.5	S	2	S
10R	*S. sanguinis*	0.5	I	≤0.12	S	≤2	S	≤0.25	S	≤0.06	S	–	–	0.5	S	2	S
11R	*S. sanguinis*	0.5	I	≥8	R	≤2	S	≥16	R	≤0.06	S	–	–	0.5	S	2	S
17R	*S. sanguinis*	≤0.25	S	≤0.12	S	≤2	S	0.5	S	≤0.06	S	–	–	0.5	S	2	S
19R	*S. sanguinis*	≤0.25	S	≤0.12	S	≤2	S	≤0.25	S	≤0.06	S	–	–	0.5	S	2	S
23R	*S. sanguinis*	2	I	≥8	R	≤2	S	≥16	R	≤0.06	S	–	–	0.5	S	2	S
12R	*S. gordonii*	≤0.25	S	≤0.12	S	≤2	S	0.5	S	≤0.06	S	–	–	0.5	S	2	S
22R	*S. gordonii*	≤0.25	S	≥8	R	≤2	S	≥16	R	≤0.06	S	–	–	0.5	S	2	S
15R	*E. faecalis*	≤2	S	2	I	2	S	≤1	S	≤0.12	S	4	R	1	S	–	–
15R	*E. casseliflavus*	≤2	S	0.5	S	1	S	≤1	S	≤0.12	S	1	R	2	R	–	–
29R	*E. casseliflavus*	≤2	S	2	S	2	S	≤0.5	S	≤0.12	S	1	R	2	R	–	–

*Note*: Numerical values are expressed in μg/mL.

Abbreviations: I, Intermediate; Inter, interpretation of MIC according to CLSI; MIC, minimum inhibitory concentration; R, resistance; S, sensitive.

### Metagenomic analysis of patients' samples

#### Community microbiome

Shotgun metagenomic sequencing was performed on the 32 samples. Patients were grouped into two cohorts: symptomatic (*n* = 19) and asymptomatic (*n* = 13) and analyzed by the CosmosID Hub.

**FIGURE 1:**
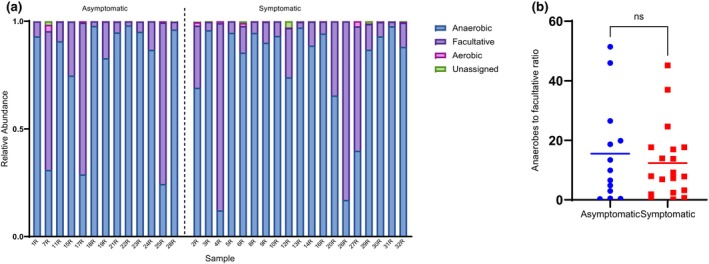
Relative abundance of bacteria (identified by shotgun metagenomics) based on their aerotolerance. (a) Relative abundance of bacteria based on their aerotolerance in each study sample. (b) Statistical analysis of anaerobes to facultative anaerobes ratio in each sample between the asymptomatic and symptomatic groups showed no difference between the two groups.

**FIGURE 2:**
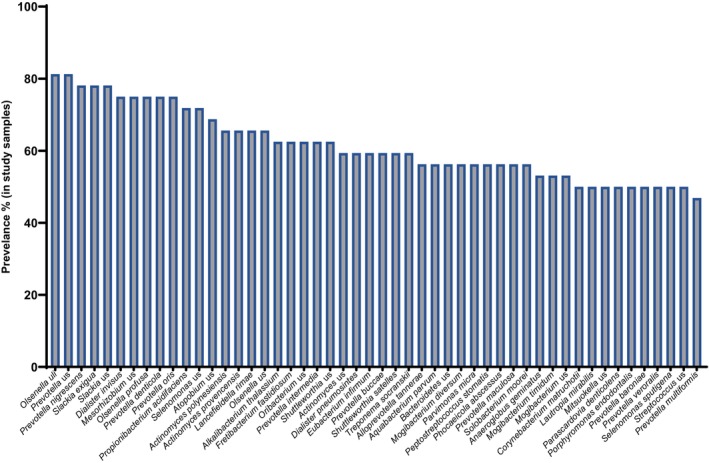
Prevalence (%) of bacteria in study samples at species level for the top 50 bacteria detected by shotgun metagenomic sequencing. us denotes unassigned species.

Bacterial communities in the two cohorts comprised 12 phyla (Figure S2). A total of 366 different bacterial species were detected across the two cohorts. Figure S3 is a species‐level heatmap clustering both samples and species and shows the top 50 most abundant species. Intermixing of the groups shows a lack of clear trends in community composition changes based on symptoms. The species were assigned into 11 bacterial phyla and 1 archaeal phylum (1 species, *Methanobrevibacter oralis*). The latter was found in 3 samples, albeit at a low abundance. The major phyla of all species included *Firmicutes* (131 species), *Proteobacteria* (72 species), *Actinobacteria* (69 species) and *Bacteroidetes* (63 species). Of the 366 species, 179 (48.91%) were anaerobic bacteria, and 164 (44.81%) were facultative anaerobes (Figure S4). Aerobic bacteria accounted for 2.73% of the whole bacterial population. This results in an anaerobic:facultative:aerobic distribution ratio of 1:0.9:0.06. When studying the bacterial distribution based on aerotolerance in each cohort (symptomatic and asymptomatic), the same trend was observed: 1:0.9:0.07 and 1:0.9:0.04, respectively. Analysis of the architecture of each sample based on constituent taxa aerotolerance showed that the majority of samples (81.3%; 26 out of 32) had a high anaerobe‐to‐facultative anaerobe ratio, ranging from 1.9 to 51.4 (Figure 1). No significant difference in the anaerobe‐to‐facultative anaerobe ratio between the asymptomatic group and the symptomatic group was found (Figure 1). The prevalence of genera in the study samples, regardless of symptom status, was calculated (Figure S5) and showed that *Actinomyces* and *Olsenella* had the highest prevalence, each at a rate of 87.5%. *Prevotella* was detected at a rate of 84.38%, while both *Slackia* and *Dialister* were detected at a rate of 78.13%. *Streptococcus*, *Lactobacillus* and *Enterococcus* were found in 62.5%, 50% and 40.6% of the samples, respectively. At the species level, *Olsenella uli* and *Prevotella* us (unassigned species) were most prevalent, at a rate of 81.25%, with a relative abundance that ranged from 0.21% to 20.26% and 0.04% to 13.86%, respectively (Figure 2). This was followed by *Prevotella nigrescens*, *Slackia exigua* and *Slackia* us (unassigned species) at a prevalence of 78.1%. *Dialister invisus*, *Olsenella profuse*, *Prevotella denticola* and *Prevotella oris* were all detected at a 75% prevalence. Several *Enterococcus* species were identified, each at a different prevalence rate. *Enterococcus casseliflavus* and *Enterococcus italicus had* a prevalence of 12.5% and 15.6%, respectively, while both *E. faecalis* and *E. faecium* had a prevalence of 3.1%. Several unassigned species of *Enterococcus* were detected with a prevalence of 34.4%. *Porphyromonas gingivalis* was detected at a 28.13% prevalence rate with a relative abundance that ranged between 0.08% and 53.68%.

Alpha‐diversity indices (Chao1, Simpson and Shanon diversity indices) showed no significant difference in species richness and evenness between the two cohorts (Wilcoxon rank‐sum, *p* value = .89, .65 and .50, respectively) (Figure S6a). Beta diversity assessed using the Bray‐Curtis index and visualized by PCoA revealed no significant compositional differences between cohorts (Permanova, *p* = .80) (Figure S6b). LEfSe analysis was undertaken to determine if any species were discriminative between the symptomatic and asymptomatic groups. The LEfSe plot showed enriched taxa with LDA threshold scores >2, which were considered significant. *Prevotella marshii* and *Peptoamaerobacter stomatis* were more enriched in the symptomatic cohort, while *Megasphaera elsdenii*, *Actinomyces polynesiensis*, *Enterococcus* spp., and *Rothia dentocariosa* were more differentially abundant in the asymptomatic cohort (Figure S6c).

#### Resistome

**FIGURE 3:**
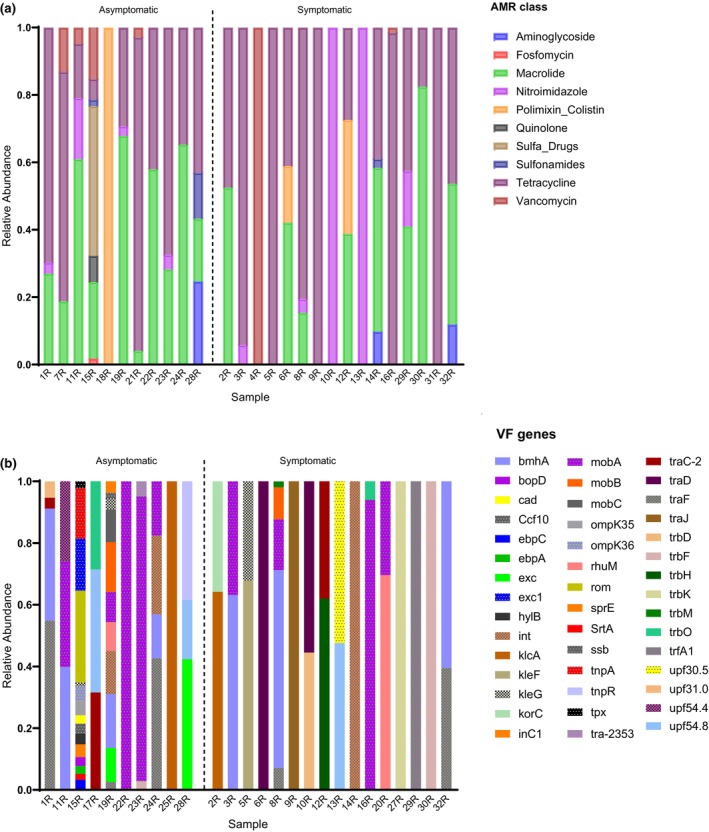
Resistome and virulome in each patient's sample identified by shotgun metagenomics. (a) Composition and relative abundance of antimicrobial resistance (AMR) for antimicrobial classes in each patient sample detected by shotgun metagenomic sequencing. (b) Composition and relative abundance of virulence factor (VF) genes in each patient sample detected by shotgun metagenomic sequencing.

Antibiotic resistance genes were found in 27 samples (84.4%, *N* = 32). A total of 59 antimicrobial resistance (AMR) genes were identified, including polymyxin‐colistin (Figure S7). However, since different AMR genes might encode for the same antibiotic resistance, the relative abundance of AMR genes was aggregated to the corresponding antimicrobial class level for each sample (Figure 3a).

AMR genes for tetracyclines, macrolide, and nitroimidazole were most abundant. Resistance to polymyxin‐colistin was found in 3 samples (6R, 12R and 18R). In sample 18R, polymyxin‐colistin was the only AMR gene identified; however, in samples 6R and 12R, resistance to two other antibiotic classes, namely macrolide and tetracycline, was identified. Of the 27 samples, 23 had tetracycline AMR genes, while 18 samples had macrolide resistance genes. Nine of the samples had nitroimidazole resistance genes, and six had vancomycin resistance genes, while the fosfomycin resistance gene was only found in sample 15R and in low abundance. In general, genes encoding tetracycline resistance were the most abundant in all samples. Alpha and beta analyses showed no significance between cohort groups (Figure S8). LEfSe analysis did not change any discriminative features between the two cohort groups.

#### Virulome

Virulence genes (*n* = 82; Figure 3b) were found in 26 samples (81.3%, *n* = 32). The Virulence Factors Database (VFDB) was used for gene classification. Three genes (*ebpA, ebpC, and strA*) were adhesion‐associated, while *Hylb* and *traJ* were genes associated with invasion, and *bopD* was associated with biofilm formation. Classification of other genes was unsuccessful; however, review of the literature showed that other genes related to immune modulation (*omp* genes), conjugation, regulation and protein mobilization (*mob* genes).

Alpha diversity of the virulome was calculated to assess richness and evenness using Chao1, Simpson and Shannon diversity indices. Statistical analysis showed that there was a significant difference in the alpha diversity between the two infection cohort groups (Wilcoxon rank‐sum, *p* value = .01, .02, and .02, respectively). Although cohort group differences were not found following beta diversity analysis (Permanova, *p* value = .78) (Figure S9), LEfSe analysis showed that no specific virulence gene was enriched or differentially abundant in the two cohort groups.

#### Functional potential analysis

Functional profiling was performed to assess the metabolic activities and functional characteristics of apical periodontitis microbial communities and to identify a possible signature of these communities based on symptoms.

**FIGURE 4:**
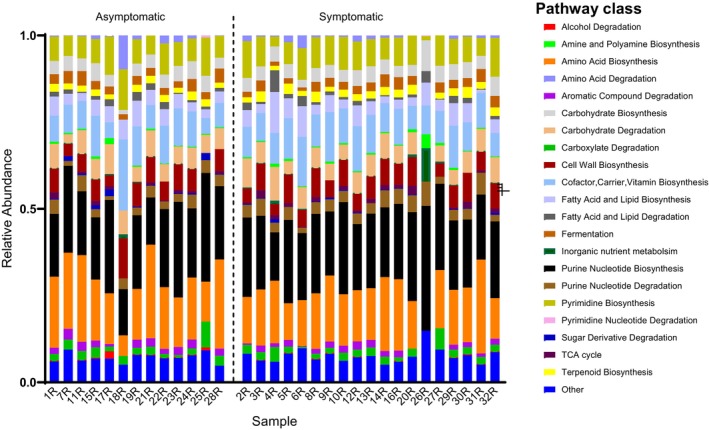
Relative abundance of functional pathway classes in each patient's sample detected by shotgun metagenomic sequencing.

A total of 286 MetaCyc pathways were identified. Alpha and beta diversity analyses showed no differences in functional profiles between the two cohort groups (Figure S10) and LefSe analysis showed no specific pathway was enriched in any of the cohort groups. Pathways were manually annotated to their pathway class using MetaCyc. Figure 4 shows the functional pathway classes of both cohort groups. The 21 identified pathways were involved in core cellular functions including nucleotide, amino acid, carbohydrate, fatty acid and lipid metabolism. Biosynthesis pathways for cofactors, carriers and vitamins included those involving heme, thiamine, cobalamin, biotin, vitamin B6 and folate. Fermentation pathways included those of pyruvate and short fatty acids. Four pathways associated with terpenoid biosynthesis were also detected at a prevalence of 96.9%.

### Bacterial composition of developed biofilms

Biofilms were formed on HA coupons for 72 h under anaerobic conditions from samples 8R and 15R to represent those from symptomatic and asymptomatic apical disease, respectively. Selection of these samples was based on sequencing and culture results, as these two samples had high alpha diversity by metagenomic analysis (Figure S11). In addition, the two samples had the highest bacterial diversity by culture, with 15R yielding 4 different culturable genera and 8R yielding 6 culturable genera (Table 1).

#### Bacterial composition of developed biofilms using culture‐dependent techniques

**FIGURE 5:**
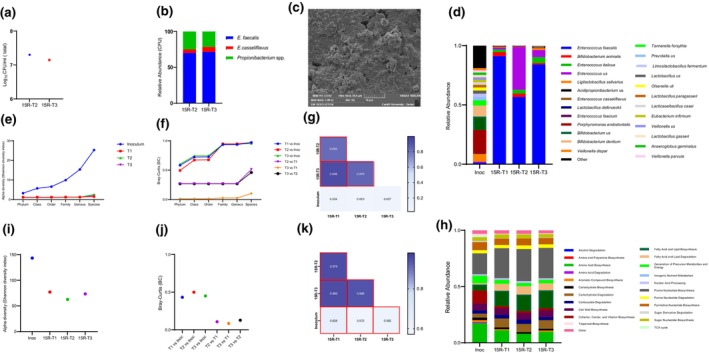
Bacterial composition and functional profile of biofilm generated from patient's sample (15R) identified by culture‐dependent technique and shotgun metagenomics. (a) The number of colony forming units (CFU) of two replicates (T2, T3) of biofilms developed on HA coupons for 72 h using sample 15R as seeding inoculum. (b) Stacked bar plots show the relative abundance of culturable species in each replicate of 15R developed biofilms. (c) Scanning electron microscopy (SEM) of developed biofilm. (d) Bacterial composition at species‐level of 15R inoculum and developed biofilms using metagenomic sequencing. Inoculum (Inco) bar plot represents average data generated from two inoculum that were taken from patient's sample (15R) on different days. Biofilm bar plots (15R‐T1, 15R‐T1, 15R‐T3) represents data generated from biofilms developed in three independent experiments. (e) Alpha diversity indices at each taxonomic level for inoculum (Inoc) and developed biofilm replicates (T1, T2, T3). (f) Bray–Curtis (BC) dissimilarities at each taxonomic level between inoculum (Inco) and biofilms replicates (T1, T2, T3) and among biofilm replicates. (g) Pairwise correlation coefficients of species‐level relative abundance between inoculum and biofilms replicates and among biofilm replicates. Correlation coefficient values are shown in the squares. Statistically significant pairs (*p* < .05) are shown in red squares. (h) Functional pathway classes composition of 15R inoculum versus developed biofilms. (i) Alpha diversity indices of functional pathways for inoculum (Inoc) and developed biofilm replicates. (j) Bray‐Curtis (BC) dissimilarities of functional pathways between inoculum (Inco) and biofilms replicates and among biofilm replicates. (k) Pairwise correlation coefficients of functional pathways between inoculum and biofilms replicates and among biofilm replicates. Correlation coefficient values are shown in the squares. Statistically significant pairs (*p* < .05) are shown in red squares.

**FIGURE 6:**
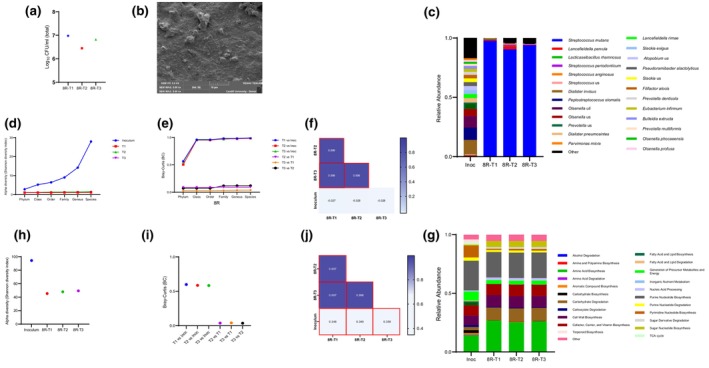
Bacterial composition and functional profile of biofilm generated from patient's sample (8R) identified by culture‐dependent technique and shotgun metagenomics. (a) The number of colony forming units (CFU) of three replicates (T1, T2, T3) of biofilms developed on HA coupons for 72 h using sample 8R as seeding inoculum. (b) Scanning electron microscopy (SEM) of developed biofilm. (c) Stacked bar plots showing relative abundance of bacterial composition at species level of 8R inoculum versus developed biofilms using metagenomic sequencing. Inoculum (Inoc) barplot represents average data generated from two inoculum that were taken from patient's sample (8R) on different days. Biofilm barplots (8R‐T1, 8R‐T1, 8R‐T3) represents data generated from biofilms developed in three independent experiments. (d) Alpha diversity indices at each taxonomic level for inoculum (Inoc) and developed biofilm replicates (T1, T2, T3). (e) Bray–Curtis (BC) dissimilarities at each taxonomic level between inoculum (Inoc) and biofilms replicates (T1, T2, T3) and among biofilm replicates. (f) Pairwise correlation coefficients of species‐level relative abundance between inoculum and biofilm replicates and among biofilm replicates. Correlation coefficient values are shown in the squares. Statistically significant pairs (*p* < .05) are shown in red squares. (g) Stacked bar plots showing relative abundance of functional pathway classes of 8R inoculum versus developed biofilms. (h) Alpha diversity indices of functional pathways for inoculum (Inoc) and developed biofilm replicates. (i) Bray–Curtis (BC) dissimilarities of functional pathways between inoculum (Inco) and biofilms replicates and among biofilm replicates. (j) Pairwise correlation coefficients of functional pathways between inoculum and biofilm replicates and among biofilm replicates. Correlation coefficient values are shown in the squares. Statistically significant pairs (*p* < .05) are shown in red squares.

Biofilm developed from sample 15R produced three distinct colony types with a total mean log_10_CFU/mL count of 7.2 (SD ± 0.1) (Figure 5a). Differences in colony characterization aided the determination of the relative abundance of each species. *Enterococcus faecalis* was the predominant species identified by culture‐dependent approaches, with a mean relative abundance of 70.7% (SD ± 1.0) (Figure 5b). While *Propionibacterium* spp. and *E. casseliflavus* relative abundances were 23.2% (SD ± 2.5) and 6.1% (SD ± 1.5), respectively. The relative abundance of species from the 15R biofilm using a culture‐dependent approach was similar for the two biological replicates (Figure 5b). Sample 8R biofilms produced a total mean log_10_CFU/mL of 6.8 (SD ± 0.3) (Figure 6a) with at least two species, namely *S. anginosus* and *Lactobacillus gasseri* as biofilm constituents. However, the relative abundance of each species based on culture‐dependent techniques was not determined due to difficulties in discriminating these species from colony appearance. Figures 5c and 6b show SEM images of 72 h old biofilms developed on HA coupons using 8R or 15R as the seeding inocula.

#### Bacterial composition of developed biofilms and functional potential analysis using shotgun metagenomics

Shotgun metagenomic sequencing was performed on the inoculum and developed biofilms to measure their community architecture (constituent taxa and their relative abundances) and reproducibility of the *ex vivo* biofilm communities. Sample 15R contained 114 species, with *Porphyromonas endodontalis* the most predominant (19.9% mean relative abundance) (Figure 5d). *Meanwhile*, biofilms derived from this sample contained 9 species, with *E. faecalis* being the predominant species, with 77.3% mean relative abundance (Figure 5d). Comparison of alpha and beta diversity between the 15R sample inoculum and its developed biofilms, categorized by taxon, revealed the highest resemblance at the phylum level. However, this similarity progressively decreased as the taxonomic resolution increased, reaching its lowest point at the species level (Figure 5e,f). This pattern was the same for the 8R sample inoculum and its developed biofilm (Figure 6d,e). Stacked bar plots of sample 8R bacterial composition showed that it was more diverse (105 species) than its derived biofilm (12 species) (Figure 6c). *Dialister invisus* was most predominant in the sample, while *S. mutans* was most predominant in the developed biofilm, with a mean relative abundance of 12.1% and 94.0%, respectively (Figure 6c). Pearson correlation was used to determine resemblance between the inoculum composition and the developed *ex vivo* biofilms. The correlation between each inoculum and the corresponding biofilm was neither strong nor significant (Figures 5g and 6f).

The community architecture of developed biofilms biological replicates was investigated to address the reproducibility of *ex vivo* biofilms. Figures 5d and 6c show resemblance in bacterial community architecture among the biological replicates for both samples, which was indicative of a high degree of reproducibility among biological replicates. To further test reproducibility, the Pearson correlation was used to determine correlations between the biological biofilms' replicates. The biological biofilm replicates from each sample showed a very strong significant positive correlation with Pearson correlation coefficients ranging between 0.852–0.998 and 0.998–0.999 for pairs of biofilms biological replicates that were derived from 15R or 8R inoculum, respectively (Figures 5g and 6f).

Stacked bar plots of functional profiles of the inoculum and corresponding developed biofilms (Figures 5h and 6g) showed less variance compared to their taxonomic profiles. Alpha and beta diversity comparisons are presented in Figures 5i,j and 6h,i. Pearson correlation analysis revealed a positive and significant correlation between the inoculum and developed biofilms, with correlation coefficients ranging between 0.526 and 0.592 for the 15R sample, and 0.348 to 0.350 for 8R. Meanwhile, a very strong, significant positive correlation was observed between the biological biofilm replicates from each sample (Figures 5k and 6j).

## DISCUSSION

Primary endodontic infections are typically polymicrobial, and no single species is specifically associated with the infection (Neelakantan et al., 2017). Previous studies have characterized endodontic infections from specific parts of the world, but the Arabian Gulf region has not been among these. An aim of this present study was, therefore, to address this knowledge gap by characterizing the microbiota of primary endodontic infections in the Arabian Gulf region.

Studies have investigated the microbiota of endodontic infections, primarily using culture‐dependent techniques, targeted molecular methods, or 16S rRNA gene sequencing (Rôças et al., 2011; Wong et al., 2021). Given the recognized limitations of these approaches, the current study used both metagenomic sequencing and culture‐dependent techniques. Metagenomic sequencing has the advantage of defining not only bacterial composition but also other domains (Breitwieser et al., 2019; Weinstock, 2012). Furthermore, metagenomic sequencing can establish the presence of resistance and virulence genes, as well as the predicted functional profile associated with microbial communities. This information can be used to assess the effects of clinical practices and inform future approaches (Bowers et al., 2017; Breitwieser et al., 2019). Metagenomic data can be supported by a culture‐based approach, as the latter is indicative of viability.

The findings of this study, which included samples from the root canals of necrotic teeth with apical disease, confirmed the polymicrobial nature of endodontic infections (Jhajharia et al., 2015; Neelakantan et al., 2017). Shotgun metagenomic analysis showed that bacteria were the predominant microbial component with an absence of fungal and viral species (except for bacteriophages‐data not shown). Compared with the metagenomic method, a considerable number of species were not identified by the culture‐dependent technique. This was likely due to culture limitations and challenges when distinguishing between different species based on colony appearance (Franco‐Duarte et al., 2019; Rajapaksha et al., 2019). The metagenomic method may have also detected DNA from bacteria that were historically present in the infection.

The microbial composition of root canal infections evolves with time, with facultative bacteria being the most abundant in the early phases of infection (Wong et al., 2021). However, as infection progresses, the low oxygen tension and depletion of nutrients lead to an ecology that favours anaerobic microorganisms (Narayanan & Vaishnavi, 2010; Wong et al., 2021). In this study, metagenomic analysis showed that anaerobic bacteria were the major constituents of the taxa architecture in the majority of samples, and this was consistent with previously reported studies (Munson et al., 2002; Rôças & Siqueira, 2008; Siqueira Jr & Rôças, 2005).

A recent systematic review of the microbiota of root canal infections using Next‐Generation Sequencing (NGS) showed the most abundant phyla were *Firmicutes*, *Bacteroidetes*, *Proteobacteria*, and *Actinobacteria* (Manoil et al., 2020), which is in agreement with the findings of this present study. *Olsenella uli*, *Prevotella* spp., *Slackia* spp. and *Dialister invisus* had the highest prevalence rates in the current study as reported previously (Bouillaguet et al., 2018; Narayanan & Vaishnavi, 2010). *Prevotella* and *Olsenella* are anaerobic bacteria and among the most predominant genera reported in a recent study using 16S rRNA sequencing (Nardello et al., 2020). Although the aforementioned study only used five patients, an important strategy introduced in that research was the use of RNA‐based NGS rather than DNA‐based NGS. This approach enabled the identification of the activity of uncultivatable or difficult‐to‐culture bacteria, which is a limitation of DNA‐based NGS.

Gram‐positive facultatively anaerobic bacteria of the *Enterococcus* genus, specifically *E. faecalis*, have been associated with persistent and secondary endodontic infections (Barbosa‐Ribeiro et al., 2021; Wong et al., 2021). In the current study, the prevalence rate of *Enterococcus* detected by shotgun metagenomic was 40.6%, but these were in relatively low abundance. However, the genus was detected at a lower rate by culture. The discrepancy between metagenomic analysis and culture could be attributed to the ability of *Enterococcus* to occur in a viable but nonculturable (VBNC) state. Furthermore, as mentioned earlier, metagenomic analysis may also have detected historical DNA presence. As metagenomic sequencing detects sequences of all nucleic acids in a specimen, it does not distinguish between live and dead cells (Gaca & Lemos, 2019; Weinstock, 2012). A previous study of the microbiota of primary and secondary apical periodontitis showed *E. faecalis* to be the most abundant species detected in secondary apical periodontitis, with higher proportions than in primary infections (Bouillaguet et al., 2018).

The severity of symptoms of endodontic infections and response to treatment likely relates to the constituent species, their relative abundance and interactions (Siqueira Jr. & Rôças, 2022; Siqueira & Rôças, 2013). Several studies have shown differences in the community structure of symptomatic and asymptomatic infections (Rôças et al., 2011; Sakamoto et al., 2006; Santos et al., 2011). To address this aspect, the patients' samples in this study were categorized into two groups according to symptoms. Alpha and beta diversity analysis showed no differences between the microbial communities of the two infection groups. The presence of a pathogen in a community does not necessarily equate to pathogenicity, as other factors also contribute (Peterson, 1996; Siqueira, 2002; Siqueira & Rôças, 2013). The presence of bacteria at an infective dose is a major contributor in determining bacterial pathogenicity (Peterson, 1996; Siqueira, 2002; Siqueira & Rôças, 2013). Bacterial load has a direct impact on virulence and the emergence of symptoms. Therefore, LEfse analysis was used to identify discriminative features between the two cohorts and showed that the anaerobic bacteria *Prevotella marshii* and *Peptoanaerobacter stomati*s were most prevalent and in higher relative abundance in the symptomatic cohort, whereas *Megasphaera elsdenii*, along with three other species, was differentially abundant in the asymptomatic cohort. Using 16S RNA sequencing, Sakamoto et al. (2006) also found that *Megasphaera* spp. were present only in asymptomatic cases, while *Prevotella intermedia* was only detected in symptomatic cases. Despite these results, confirming the positive association between candidate endodontic pathogens and symptoms could benefit from a larger sample size and the use of RNA‐based metagenomic sequencing.

The virulence factors that species possess are clearly important in the progression of infection (Peterson, 1996). Virulence factors enable microorganisms to colonize, resulting in tissue damage that might eventually progress to local and systemic inflammation. Determining virulence factors helps in understanding microbial pathogenesis and also may identify suitable targets for novel drug development (Wu et al., 2008). In the current study, endodontic infections, regardless of symptom status, had various genes that regulate several virulence factors associated with adherence, biofilm formation, invasion and immune modulation. However, it must be recognized that these genes might have been differentially expressed under specific conditions.

Oral microbiota has been suggested to act as a reservoir for AMR genes, including those associated with resistance to tetracyclines and macrolides (Arredondo et al., 2021; Roberts & Mullany, 2010). Characterizing the resistome in this study showed that both symptomatic and asymptomatic samples had genes encoding resistance, with those associated with resistance to tetracyclines, macrolides and nitroimidazole being most abundant. Phenotypic resistance was evident in the tested isolates, with a high prevalence of resistance to tetracycline and erythromycin (a macrolide antibiotic). This confirms that the resistance genes detected in the metagenomic analysis were not only present but also functionally expressed, conferring actual resistance to the corresponding antibiotics. Tetracycline is a broad‐spectrum antibiotic used to treat human infection as well as to promote animal growth (Chopra & Roberts, 2001). In dentistry, tetracycline is used to treat periodontal infection and as a prophylactic antibiotic (Chopra & Roberts, 2001). This could explain the observed high diversity and prevalence of genes encoding tetracycline resistance in oral microbiota (Arredondo et al., 2021; Truong et al., 2022; Villedieu et al., 2003). This observation is consistent with the results of the current investigation, which found a high prevalence of tetracycline‐resistant genes with several modes of action (efflux pump, ribosome protection, and enzymatic modification).

Polymyxin antibiotics, polymyxin B and polymyxin E (colistin), are last‐line treatments for Gram‐negative multidrug‐resistant infections (Shatri & Tadi, 2023). Studies have shown that *pgpB* plays a role in polymyxin B resistance, and inactivation of this gene increases susceptibility to colistin (Zhu et al., 2020). The *PgpB* gene was found in three samples in the present study, and analysis of the commonality between the three samples revealed the presence of several shared genera. However, few species were shared, with *P. gingivalis* having the highest abundance in the three samples. *Porphyromonas gingivalis* is a periodontopathogen based on its association with periodontal infections (How et al., 2016). This species has also been isolated in high prevalence from endodontic infections (Barbosa‐Ribeiro et al., 2021; Siqueira et al., 2008; Zargar et al., 2020), and this was also the case in the present study. *Porphyromonas gingivalis* is known for its pronounced polymyxin B resistance (Coats et al., 2009; Díaz et al., 2015; How et al., 2016).

In addition to resistance to tetracycline and erythromycin, antibiotic susceptibility profiling showed that some of the cultivated bacteria in this study exhibited resistance to other antibiotics such as quinupristin/dalfopristin, chloramphenicol, and vancomycin. This could be clinically significant, especially in the case of dissemination, as untreated apical periodontitis can lead to severe infections that may extend beyond the oral cavity. The pathogens responsible for these infections elsewhere in the body, as shown in this study, can harbour multiple AMR genes, making intervention more challenging.

The identified AMR genes, along with the corresponding phenotypic resistance, indicate that oral microbiota exhibit resistance to antibiotics. The presence of these AMR genes may be driven by selective pressure from the misuse of antibiotics, as well as human exposure to residual antibiotics used in agriculture and the livestock industry (Llor & Bjerrum, 2014; Manyi‐Loh et al., 2018). Such selective pressures contribute to the emergence of resistance (Kolář et al., 2001). To fully understand the root causes of antibiotic resistance in the oral cavity, further research is needed from a One Health perspective, which considers the interconnectedness of human, animal, and environmental health. This also underscores the importance of controlled antibiotic prescription in dental cases. As highlighted in a recent editorial paper, it is important to reiterate that antibiotics are not the first‐line therapy for most dental issues and should be reserved for specific clinical scenarios (Ali, 2024).

To detect a possible biomolecular signature associated with these infections according to symptoms, functional analysis was also performed. However, no difference in the functional profile was found between the two patient cohort groups. The identified pathways generally related to basic cellular metabolism. Nonetheless, pathways associated with terpenoid biosynthesis were detected in 96.9% of the samples. Although various terpene biosynthesis pathways in bacteria have been identified, only a few have been characterized and are not fully understood (Boronat & Rodríguez‐Concepción, 2015). Microbial terpenoids have been associated with the characteristic odour of moist soil and associated with *Actinomycetes* (Dairi, 2005). Some terpenoids that were primary and secondary metabolites have shown antibacterial and antifungal activity (Rudolf et al., 2021). This might suggest a form of antagonistic interaction might occur in the polymicrobial communities studied. Furthermore, these metabolites also were reported to have immunosuppressive activity (Rudolf et al., 2021). However, bacterial terpenoids and their role in virulence and pathogenicity do require further investigation. Despite these results and the lack of differences between symptomatic and asymptomatic groups, future work should consider a larger sample size and perform sample size calculations based on these data to allow for more robust conclusions, particularly in relation to microbial composition and clinical manifestations.

Developing *ex vivo* biofilm models that better reflect the *in vivo* situation is an active area of research. Such models would assist development of experimental approaches concerning infections and pathogen eradication. Several studies have used oral samples such as saliva as the seeding inoculum to develop *in vitro* oral biofilms (Edlund et al., 2013; Simon‐Soro et al., 2022). It was suggested that such models, referred to as ‘*ex vivo* models’, might better reflect the *in vivo* condition than use of pure cultures of preselected bacterial species (Simon‐Soro et al., 2022). There is certainly a pressing need for a common standardized biofilm model system which represents the endodontic infection microbiota and is capable of capturing the polymicrobial nature and complexity of such communities (Swimberghe et al., 2019).

Metagenomic analysis of the inoculum and developed biofilms in this current study showed that biofilms were polymicrobial communities, with sample 15R containing 9 species and 8R retaining 12 species. However, the microbial diversity was less compared to the inoculum. Metagenomic analysis showed that the microorganisms in the developed biofilms were similar to those determined by culture of the inoculum, suggesting that discrepancies in diversity between inoculum and biofilm communities could relate to the ‘seeding’ inoculum containing non‐viable or unculturable species that would not establish in the developed biofilms. It was also found that the biofilm architecture (relative abundance of detected species) was different from the inoculum, as species that were highly abundant in biofilms were in low abundance in the seeding inoculum. This could be attributed to the different growth rates of species in *in viv*o and *in vitro* environments or the inability of some species to bind to the supporting substrate for the biofilm. The conditions used in these experiments, such as the time at which biofilm was propagated, may also contribute to the observed discrepancy. Since biofilm growth is dynamic, developing *in vitro* and *ex vivo* models could benefit from temporal monitoring of the communities to identify their climax state of growth. The medium used in this research might not have supported the growth of all species. Hence, follow‐up experiments using different conditions could further help develop models that capture not only the microbial composition but also the architecture of such infections. Another consideration could be the use of dentine discs as a biofilm formation substrate to better mimic the *in vivo* situation. However, an appropriate sterilization method should be carefully chosen to preserve the *in vivo* characteristics and dentine components.

Reproducibility is a threshold requirement for any experimental model (Cheng et al., 2022). Hence, the degree of biological reproducibility in the community composition of developed biofilms using both metagenomic analysis and a culture‐dependent approach was measured. The developed biofilms showed a high degree of reproducibility among biological replicates, and this could enable their use as an experimental model to test the antimicrobial activity of current treatments or new potential treatments.

## CONCLUSION

For the first time, this study characterized the microbiota of root canals in necrotic teeth with periapical diseases from the Arabian Gulf region using both shotgun metagenomics and culture‐dependent techniques. Shotgun metagenomics showed that root canal samples from necrotic pulp had high bacterial diversity, along with the presence of AMR and virulence genes, with no microbial compositional or functional profile differences between symptomatic and asymptomatic infections. Using culture‐dependent techniques, a high number of isolated bacteria were resistant to clinically used antibiotics.

*Ex vivo* biofilm models derived from clinical samples were successfully developed and reproducibly reflected a polymicrobial nature. These models can, therefore, be exploited in future studies, including those involving antibacterial assessment assays.

## AUTHOR CONTRIBUTIONS

**Rania Nassar:** conceptualization, data curation, formal analysis, funding acquisition, investigation, methodology, writing – original draft preparation, writing – review and editing. **Mohannad Nassar:** conceptualization, data curation, formal analysis, funding acquisition, investigation, methodology, writing – review and editing. **Lobna Mohamed:** data curation, investigation, review and editing. **Abiola Senok:** Conceptualization, funding acquisition, supervision, writing – review and editing. **David Williams:** Conceptualization, funding acquisition; supervision, writing – review and editing.

## CONFLICT OF INTEREST STATEMENT

The authors declare no conflict of interest.

## ETHICS STATEMENT

Ethical approval for the study design and protocols was reviewed and approved by the Research Ethics Committee of the University of Sharjah (reference number REC‐21‐09‐20‐03).

## Supporting information


Appendix S1.

## Data Availability

The data that support the findings of this study are available from the corresponding author upon reasonable request.
