# Supplementary material for: Characterization and ex vivo modelling of endodontic infections from the Arabian Gulf region
Source: Int Endod J. 2025 Mar 26;58(7):1091–108. doi: 10.1111/iej.14227 (PMC12160973; doi:10.1111/iej.14227)
Supplement: Supplementary file 1 — Appendix S1. [file IEJ-58-1091-s001.docx]

Supplementary Materials for

**Characterisation and *ex vivo* modeling of endodontic infections from the Arabian Gulf region**

**Figure S1** A flow chart outlining the sequential steps in the study in accordance with Preferred Reporting Items for Laboratory Studies in Endodontology (PRILE) 2021 guidelines.

| **Table S1** Summary of patient demographic and clinical information (n=32). | |
| --- | --- |
| **Number of patients** | |
| **Gender** | |
| M | 26 |
| F | 6 |
| **Mean age** | 38 years (range 19-66) |
| **Tooth type** | |
| Molar | 4 |
| Premolar | 17 |
| Anterior | 11 |
| **Symptoms** | |
| Symptomatic | 19 |
| Asymptomatic | 13 |


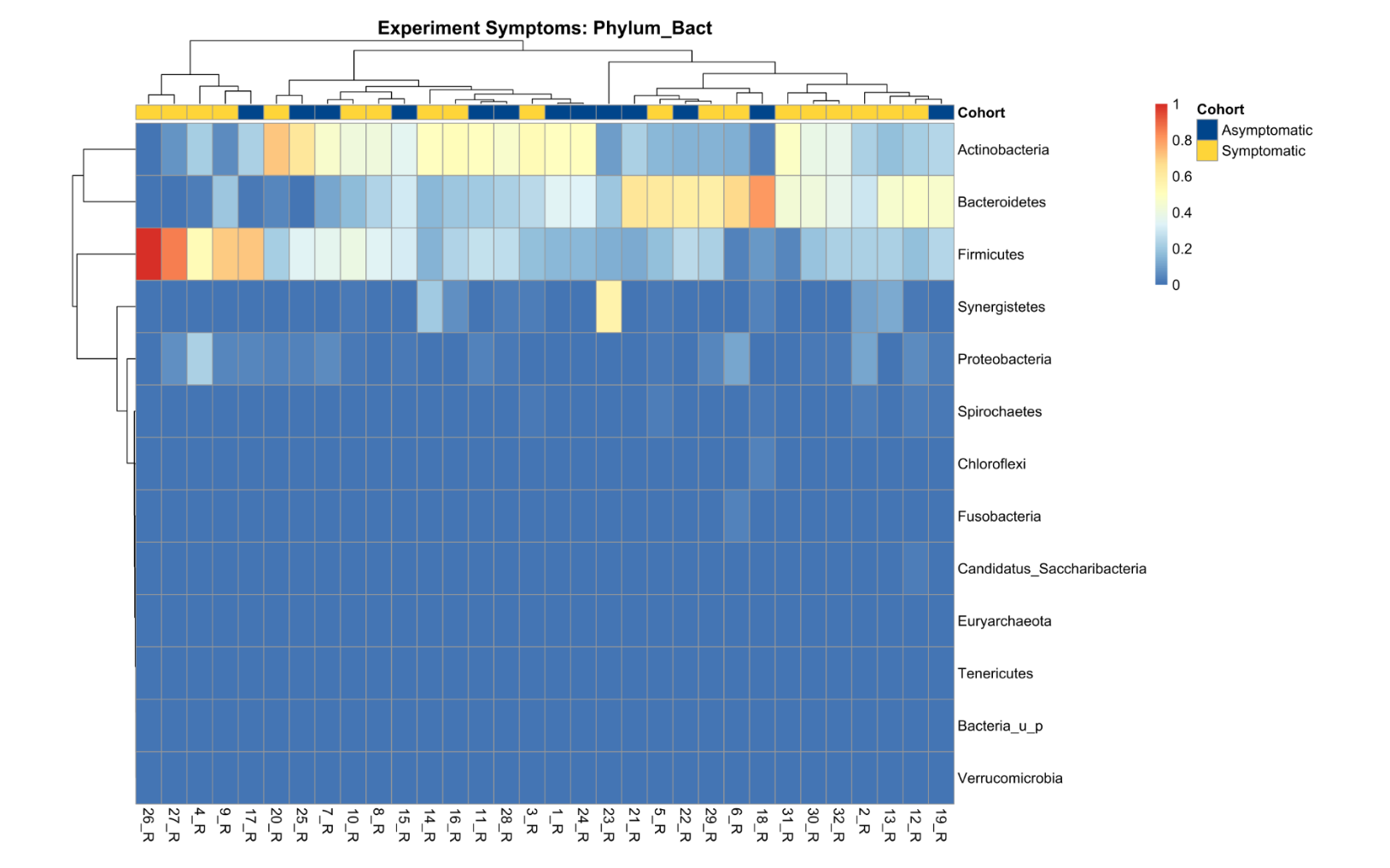


**Figure S2** Heatmap of bacterial composition identified by shotgun metagenomics. Heatmap clusters samples by community similarity at phylum level.


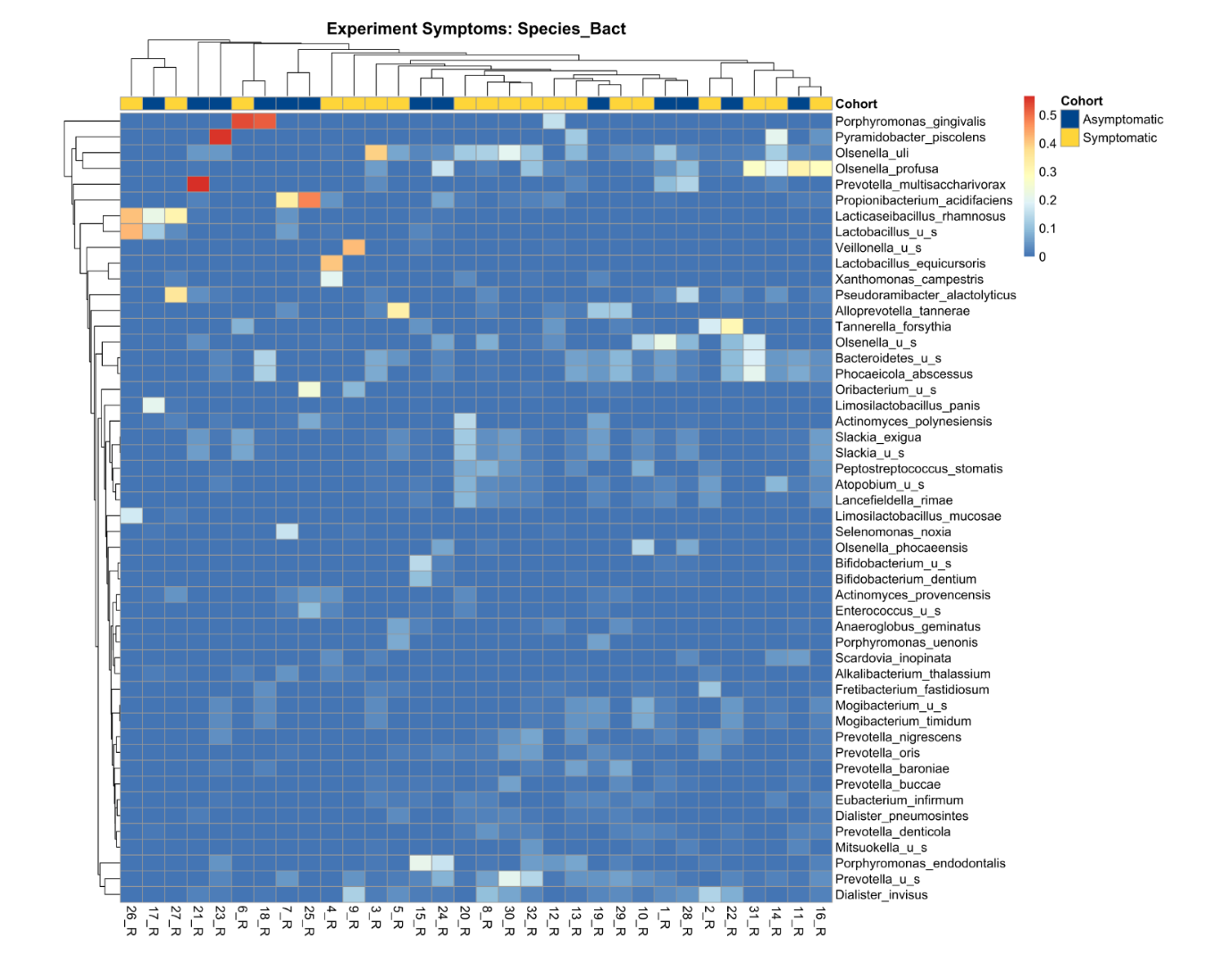


**Figure S3** Heatmap of bacterial composition identified by shotgun metagenomics. Heatmap clusters samples by community similarity and shows the 50 abundant bacteria at species level.

| a)   | b)   |
| --- | --- |

**Figure S4 Percent distribution of bacteria (identified by shotgun metagenomics) based on their aerotolerance**. a) Percent distribution of bacteria (detected in all study samples regardless of the presence or absence of symptoms) based on their aerotolerance. b) Percent distribution of bacteria in each cohort (symptomatic and asymptomatic) and based on their aerotolerance*.*

**Figure S5** Prevalence (%) of bacteria in study samples at genus level for the top 50 bacteria detected by shotgun metagenomic sequencing. ug denotes unassigned genus.

| a)   | c)   |
| --- | --- |
| b) 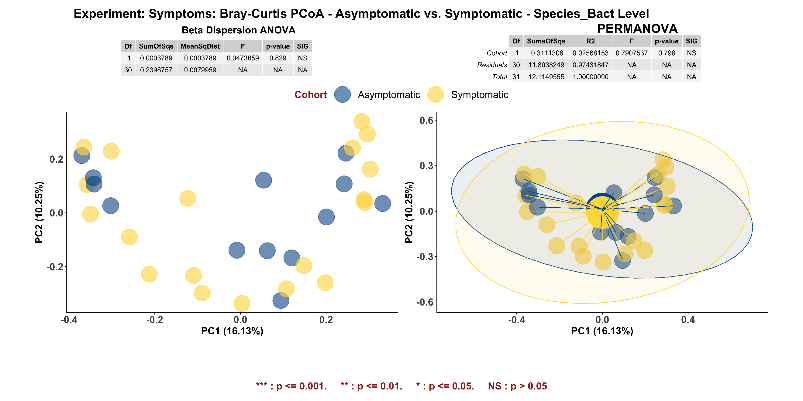 |  |

**Figure S6** **Bacterial diversity at species level among the symptomatic vs asymptomatic groups**. **a)** Alpha diversity boxplot illustrating Choa1, Shannon diversity and Simpson indices for apical periodontitis bacteria at species level among the two groups (symptomatic vs asymptomatic). Boxes represent the interquartile range between the first and third quartiles and median (internal line); whiskers denote the minimum and maximum values. **b)** Principal coordinate analysis (PCoA) of Bray-Curtis indices based on bacterial composition at species level. Each point on the plot represents an individual community’s Bray-Curtis index. The right plot shows the centroid of each group (large dot in the middle), and the lines connect each individual point belonging to that group. Ellipses represent 95% confidence intervals. The amount of variation explained by coordinates 1 and 2 is included in the axis labels. **c)** Differentially abundant bacterial species in asymptomatic and symptomatic apical periodontitis identified by LEfSe. Species were ranked by their LDA effect size. Species associated with symptomatic cases communities are shown in blue while asymptomatic are shown in red

|  |  |
| --- | --- |

**Figure S7** Composition and relative abundance of antimicrobial resistance (AMR) genes in each patient’s sample detected by shotgun metagenomic sequencing.

| a)   |
| --- |
| b) 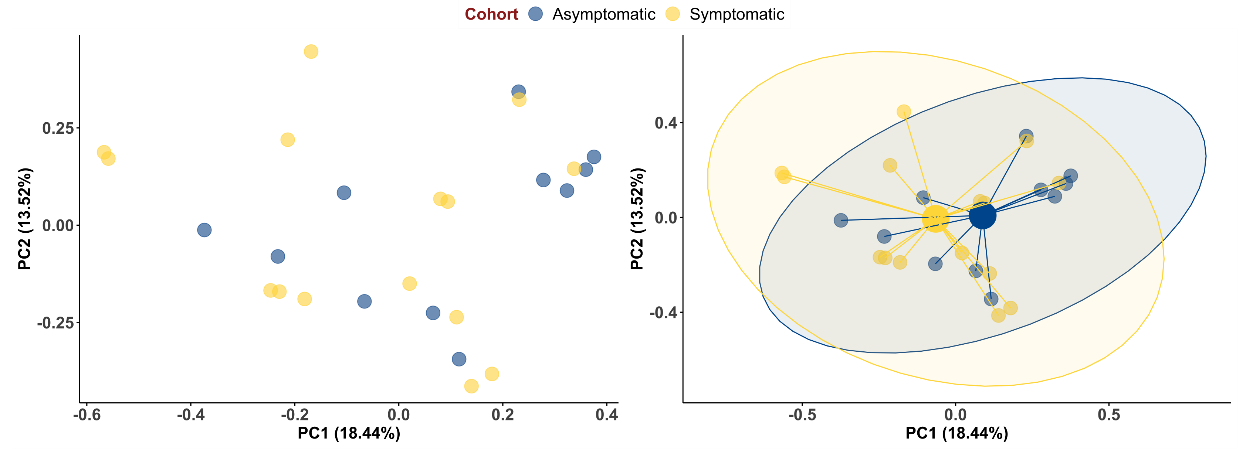 |

**Figure S8** **Alpha and beta diversity of antimicrobial resistome.** a) Alpha diversity of antimicrobial resistance genes. Boxplot shows Choa1, Shannon diversity and Simpson indices of apical periodontitis microbiota among the two groups (symptomatic vs asymptomatic). Boxes represent the interquartile range between the first and third quartiles and median (internal line); whiskers denote the minimum and maximum values. b) Principal coordinate analysis (PCoA) of Bray-Curtis indices based on virulome composition.

| a)   |
| --- |
| b)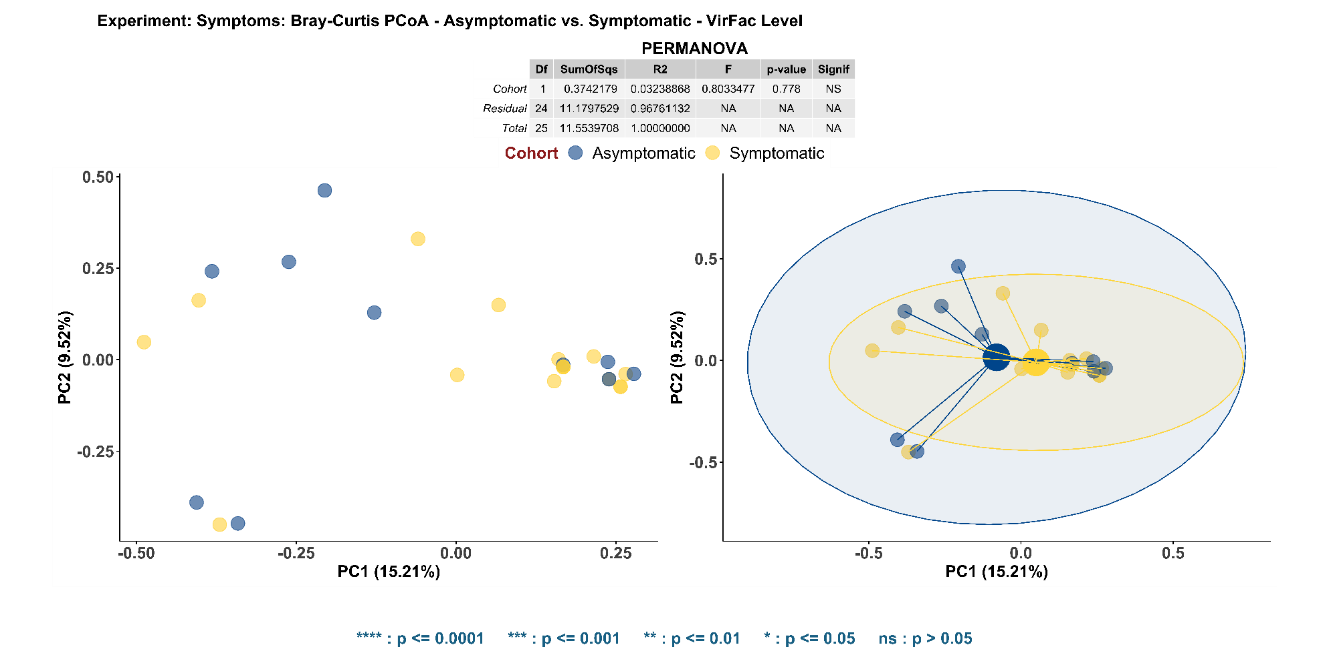 |

**Figure S9** **Alpha and beta diversity of virulome**. a) Alpha diversity of virulome. Boxplot shows Choa1, Shannon diversity and Simpson indices of apical periodontitis microbiota among the two groups (symptomatic vs asymptomatic). Boxes represent the interquartile range between the first and third quartiles and median (internal line); whiskers denote the minimum and maximum values. b) Principal coordinate analysis (PCoA) of Bray-Curtis indices based on virulome composition.

| a)  |
| --- |
| b)  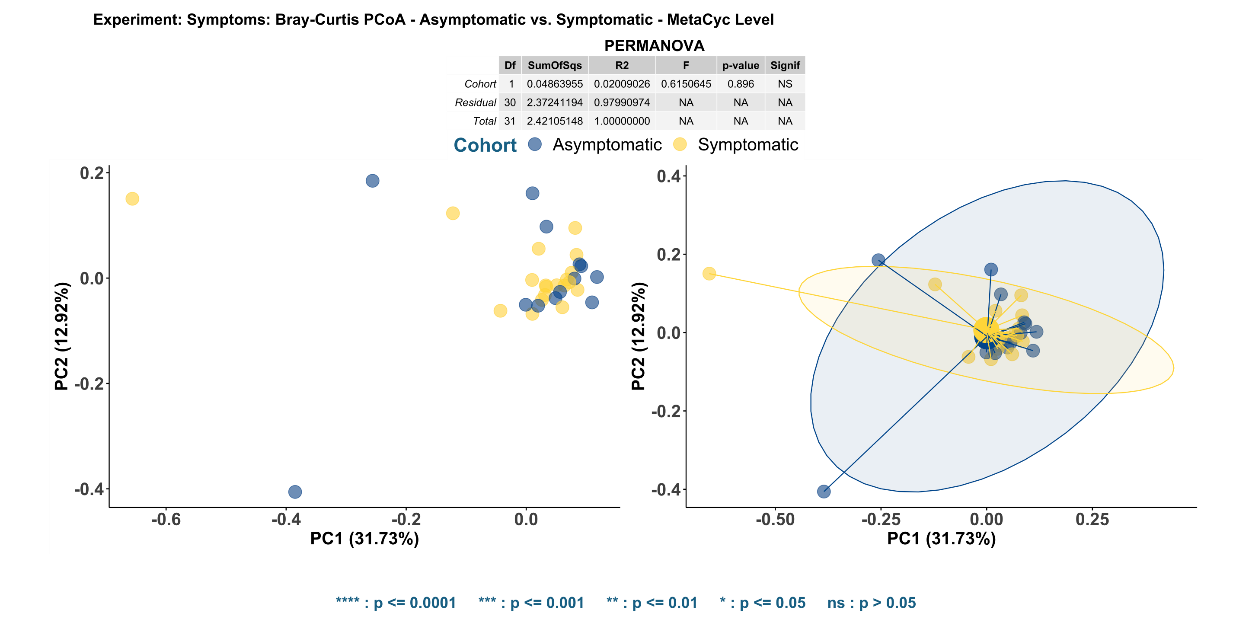 |

**Figure S10** **Alpha and beta diversity of functional profile**. a) Alpha diversity of functional profile. Boxplot shows Choa1, Shannon diversity and Simpson indices of apical periodontitis microbiota among the two groups (symptomatic vs asymptomatic). Boxes represent the interquartile range between the first and third quartiles and median (internal line); whiskers denote the minimum and maximum values. b) Principal coordinate analysis (PCoA) of Bray-Curtis indices based on virulome composition.

**Figure S11** Alpha diversity of patients’ samples at species level. Shannon diversity indices is shown. Samples 15R and 8R chosen for biofilm generation are highlighted in grey rectangle.
